# Sulcus Vocalis and Benign Vocal Cord Lesions: Is There Any Relationship?

**DOI:** 10.3390/ijerph20095654

**Published:** 2023-04-26

**Authors:** Carmelo Saraniti, Gaetano Patti, Barbara Verro

**Affiliations:** Division of Otorhinolaryngology, Department of Biomedicine, Neuroscience and Advanced Diagnostic, University of Palermo, 90127 Palermo, Italy

**Keywords:** larynx, laryngoscopic surgery, phonation, voice quality, vocal cords

## Abstract

Background: Sulcus vocalis (SV) is a longitudinal groove in the free edge of the true vocal cord. It may impair phonation with incomplete glottic closure, phonasthenia and hoarseness. This study aims to detect a correlation between benign vocal cord lesions and the incidence of the SV. Methods: A retrospective study was carried out on patients who underwent transoral surgery due to benign vocal fold lesions and were selected according to strict criteria. Patients were divided into a group with sulcus vocalis (Group wSV) and a group without sulcus vocalis (Group w/oSV). The possible correlations between variables were assessed by the Pearson chi-square test (*p* < 0.05). Results: The study included 232 vocal cord lesions in 229 patients: 62.88% were females whose mean age was 46.61 ± 14.04. The most frequent diseases were polyps (37.94%), nodules (18.53%) and Reinke’s edema (21.12%). Statistically significant relationships were found between age and SV (*p*-value 0.0005) and between mild dysplasia and SV (*p*-value 0.03). Conclusions: This study did not detect a cause–effect relationship between SV and benign vocal fold lesions. SV within vocal fold lesions is more common in younger patients, suggesting a congenital nature of SV. In conclusion, in the case of a benign vocal fold lesion, a possible SV should be considered and researched to provide the patient the best healthcare.

## 1. Introduction

The vocal fold is composed of squamous epithelium, lamina propria (superficial, intermediate and deep layers) and vocal muscle. Vocal fold lesions may lead to dysfunction due to the loss of elasticity and integrity of the vocal fold [1]. Sulcus vocalis (SV) is a longitudinal groove in the free edge of the true vocal cord; it represents an area of decreased mucosal elasticity. Indeed, this lesion changes the normal physiology of the vocal folds with impaired glottic closure, vocal fatigue, phonasthenia and hoarseness [2,3,4]. Typical dysphonia is characterized by a high-pitched and diplophonic breathy voice. Sulcus vocalis was first reported by anatomist Giacomini in 1892, who described it as an abnormality in the vocal cord [5]. Ford et al. [6] classified this lesion in three degrees of severity: type I with no functional effect and called physiological sulcus; type II, or sulcus vergeture, with a loss of the superficial layer of lamina propria; and type III, which Selleck et al. [7] consider the true sulcus vocalis, with a mucosal-loss focal area in the vocal ligament or deeper (also called pocket-type). The last two types of sulcus vocalis are considered pathological sulcus. Making the diagnosis of sulcus vocalis can be challenging. In fact, the incidence of SV is not clear and ranges from 0.4% to 48% according to various articles [3,4]. The patient should be examined by an ENT and a speech therapist to give a global approach to the disease. In fact, the vocal cords may appear normal during in-office laryngoscopy, whereas videostroboscopy can easily highlight a vocal cord lesion. Hirano et al. described usual findings during laryngoscopy: bow-shaped vocal cords with incomplete glottic closure (the so-called “spindle-shaped pattern”) and medial invaginations (almost splitting the vocal fold), a reduced mucosal wave and supraglottic hyperactivity as a compensatory mechanism [8]. However, these features can also be found in vocal cord atrophy; thus, it is important to detect the sulcus to make a differential diagnosis. In their studies, Dailey et al. and Akbulut et al. [9,10] also reported difficulties in making diagnoses of sulcus vocalis using videostroboscopy. Indeed, in cases of moderate to severe dysphonia, videostroboscopy cannot provide clear and accurate information due to failed synchronization (the fundamental frequency F0 cannot be recorded). Moreover, this exam provides qualitative data, resulting in high inter-individual variability [11]. Dailey et al. stated that SV is the most undiagnosed benign vocal cord lesion in patients examined due to benign glottic disease [9]. These drawbacks make it difficult to elaborate precise statistical studies since the diagnostic evaluation is operator-dependent. Indeed, in-office laryngoscopy provides a view tangent to SV and vocal fold lesions, resulting in difficulty to detect the sulcus itself (the authors called it the “umbrella effect”) [12].

Several therapeutic strategies, surgical and non-surgical, can be chosen since today there is no consensus about the best therapy. Voice therapy can be accepted in the first instance [13,14]; however, when the speech-therapy treatment is ineffective and functional limitations and worse quality of voice occur, a surgical treatment can be proposed: medialization laryngoplasty [15,16], pulsed laser treatment [17,18], vocal fold injections [19,20,21] or autologous tissue implantation [22,23,24,25,26,27,28,29,30,31,32,33]. To date, the latter seems to be the best therapeutic choice for the success of vocal cord flexibility and for long-term outcomes. Indeed, treatment of SV should achieve two goals: restoring the mucosal wave and fixing the anatomic abnormality.

Furthermore, sulcus vocalis seems to be correlated with coexisting vocal cord lesions. As reported in a few reviews [24,25,26,27], in cases of benign vocal fold lesions, a sulcus vocalis should always be considered since treating the benign lesion but leaving out the sulcus may not lead to the complete improvement of the patient’s symptoms, in addition to causing a predisposition to phonotrauma.

Based on these premises and on the poor literature, this study aims to evaluate the incidence of sulcus vocalis in patients undergoing transoral laryngeal surgery (TOLS) due to vocal cord pathology, seeking a correlation between the presence of a sulcus and the onset of benign vocal cord lesions.

## 2. Materials and Methods

### 2.1. Study Design

A retrospective study was carried out on patients who underwent TOLS due to benign vocal fold lesions from January 2010 to January 2020 in our Ear Nose and Throat Clinic at the University Hospital of Palermo. This study was approved by the ethical committee of our university hospital (approval number: 04/2022), and informed consent was obtained from the patients in accordance with the Helsinki Declaration.

The inclusion criteria were as follows: males and females aged between 18 and 80 years old, patients undergoing TOLS due to vocal cord lesions and histological diagnosis of a benign vocal fold lesion.

The exclusion criteria were as follows: histological diagnosis of malignant vocal fold lesions (severe dysplasia, carcinoma in situ or carcinoma) and patients undergoing TOLS due to non-vocal cord lesions.

The following data were collected: demography (age and sex), surgical technique (cold or laser surgery), histological diagnosis (nodules, polyps, Reinke’s edema, cyst, papillomatosis or others), side (right and/or left vocal cord) and number of vocal cord lesions (mono or bilateral) and presence or not and side of sulcus. Recruited patients were divided into two groups: a group of patients with sulcus vocalis (Group wSV) and a group of patients without sulcus vocalis (Group w/oSV).

### 2.2. Data Analysis

Data on the demography, surgery, vocal fold lesion and sulci of the recruited patients were collected in a data spreadsheet using Microsoft Excel, version 16.66.1. These data were reported as numbers and percentages of the total and/or mean ± standard deviation (SD). MedCalc software was used for the statistical analyses. The possible associations between the dichotomous nominal variables were assessed by calculating the Pearson chi-square tests [34]. A *p*-value of <0.05 was considered statistically significant.

## 3. Results

The study included 232 vocal cord lesions in 229 patients: 144 (62.88%) females and 85 (37.12%) males aged between 18 and 80 years old (the mean age was 46.61 ± 14.04). In particular, 77 (33.62%) patients belonged to Group wSV, and 152 (66.38%) belonged to Group w/oSV.

All patients were treated by the same surgeon (CS) to avoid biases related to different surgeons’ skills. Moreover, due to the difficulty in detecting a sulcus during an in-office laryngoscopy, we included in this study only cases where a sulcus was confirmed during the surgical procedure.

**Figure 1:**
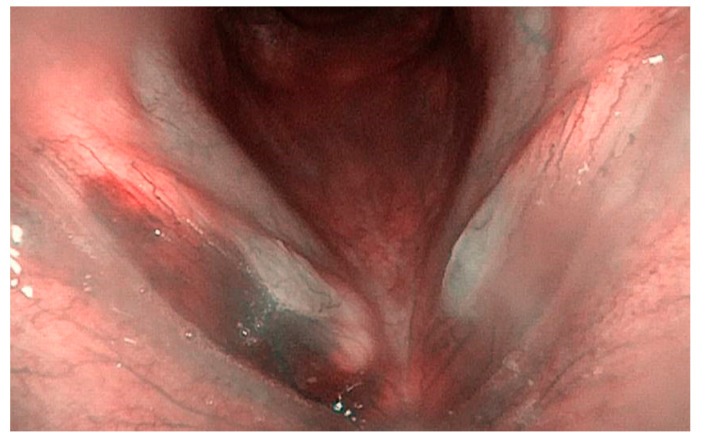
Bilateral sulcus with bilateral nodules and a right vocal cord hemorrhage due to phonotrauma (Narrow-Band Imaging).

**Figure 2:**
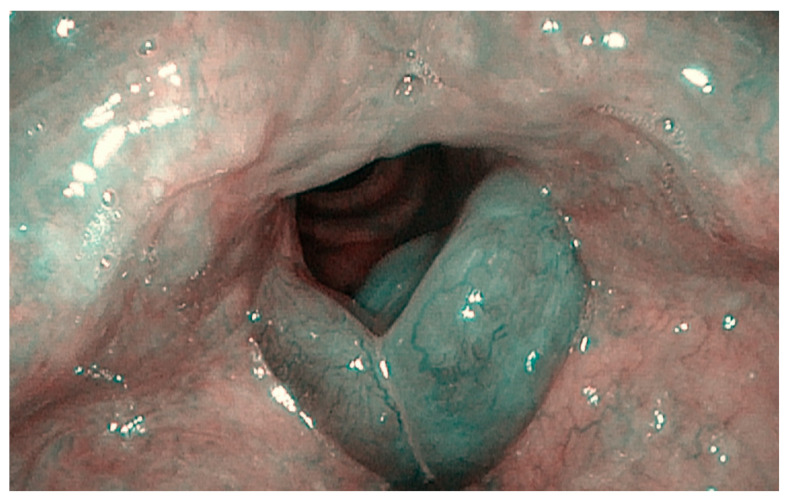
Bilateral sulcus in Reinke’s edema (Narrow-Band Imaging).

Vocal fold lesions included in the analysis were: angioma (0.43%), polyp (37.94%), nodule (18.53%), cyst (9.05), papillomatosis (0.86%), Reinke’s edema (21.12%) and mild (6.03%) and moderate (3.02%) dysplasia. The data are summarized in Table 1. The most frequent diseases were polyps (37.94%), nodules (18.53%) and Reinke’s edema (21.12%) (Figure 1 and Figure 2). In particular, as regards Group wSV, polyps were the most common finding (44.30%), followed by nodules and Reinke’s edema, which had the same incidence (20.25%). No cases of papillomatosis and/or moderate dysplasia were reported for this group.

In both groups, female prevalence was found: 67.53% for Group wSV and 60.53% for Group w/oSV. However, statistical analysis did not show any statistically significant correlation between the variable “sex” and the presence/absence of sulci (*p*-value 0.299).

Patients were divided into three age categories: (1) young people (18–44 years old), (2) middle-aged people (45–64 years old) and (3) older people (65–80 years old). The study revealed that most patients in Group wSV were young (58.44%), while 47.37% of the patients who belonged to Group w/oSV were aged between 45 and 64 years old. The chi-square test showed a statistically significant relationship between age and the presence of sulci (*p*-value 0.0005).

No association was found between the type of vocal cord lesion and the presence of a sulcus, except for mild dysplasia (*p*-value 0.03).

Also, a possible correlation between the side of the lesion and the side of the sulcus was evaluated by the chi-square test calculator for a 3 × 3 contingency table with a negative result (*p*-value 0.08) (Table 2). The SV was bilateral in 31 cases (40.26%), and 25.96% of the patients had both bilateral SV and bilateral vocal fold lesions.

## 4. Discussion

Sulcus vocalis is a longitudinal furrow that runs along the vocal cords, altering not only the vocal cords’ anatomy but their functionality, too. Vocal fold scarring means that the mucosa is tethered to the underlying tissue and cannot vibrate freely, causing vocal fatigue, incomplete glottic closure and hoarseness [1,2,3,4,6,7,9]. As written above, the vocal folds are composed of the cover (epithelium and superficial layer of lamina propria) and body (intermediate and deep layers of lamina propria and vocal muscle). As described by Hirano in 1975, according to the cover-body theory, during phonation, the airflow from the trachea leads to the vibration of the pliable cover on the body of the vocal folds [35,36]. A study by Zhou et al. demonstrated that sulcus vocalis causes a directly proportional increase in the phonation threshold pressure. This increase requires more effort to be made to provide adequate airflow. This effort results in hoarseness, vocal fatigue, phonasthenia and worse voice quality. Obviously, the effort and symptoms worsen with increasing sulcus depth [37]. As reported by Hirano et al., the vocal fatigue and hoarseness are due more to incomplete glottic closure than to stiff vocal folds [8]. Stroboscopic studies show a statistically significant increase in the mean F0 in types II and III of sulcus vocalis [38].

In 1994, Pontes et al. suggested a first classification of sulcus vocalis into the following categories: (1) sulcus stria minor, which is an epithelial depression; (2) sulcus stria major, where the mucosal invagination adheres to the deep layer, vocal ligament and muscle; and (3) pouch-shaped sulcus lesion, where the invagination results into a pouch-shaped subepithelial depression [38]. However, the most common classification was formed by Ford et al. in 1996 [6], dividing sulcus vocalis into three categories: type I, or physiologic sulcus; type II, also called sulcus vergeture; and type III, which is the “true sulcus vocalis” or a pouch-like depression [7].

The etiology of sulcus vocalis is still unclear today. The ideas of the various authors are controversial, and the scientific literature is lacking in this regard. Bouchayer et al. [1] and Ford et al. [6] ascribe these causes to gastroesophageal reflux, laryngeal chronic inflammation, errors in surgical technique, congenital alterations, mucosal atrophies, embryonic defects and others. In particular, Bouchayer et al. assumed that sulcus vocalis is the result of the rupture of an epidermoid cyst from remnants of the fourth and sixth brachial arches. According to this theory, the capsule of the epidermoid cyst has remained attached to the deep tissues of the vocal cords, resulting in the formation of an invagination that is the sulcus [1]. Moreover, Sato et al. studied the mucosa of sulcus vocalis using electron microscopy and demonstrating degenerated and fewer fibroblasts in the macula flava, resulting in changes in elastic and collagenous fibers in the vocal fold mucosa [39]. Lee et al. analyzed the histology of surgical specimens and found epithelial changes (parakeratosis, dyskeratosis and inflammatory infiltrate) in most of the cases. Based on these findings, the authors suggested a similarity with the pathogenesis of cholesteatoma [38]. Indeed, SV has a pouch-like appearance, as well as a retraction pocket of the tympanic membrane that promotes keratin accumulation and subsequent inflammation. This excessive immune response is responsible for subepithelial vocal fold damage and impaired voice quality. These epithelial changes may explain the common finding of benign vocal fold lesions with SV.

After studying four individuals of the same family with dysphonia, Martins et al. [31,32] suggested a genetic origin of sulci, such as autosomal dominant inheritance. However, the authors state that is not possible to establish a genetic transmission of this disorder.

Moreover, based on the current literature, sulcus vocalis does not seem to predominantly affect a gender or a specific age. Itoh et al. [2] studied 240 patients who were divided into three groups: (1) sulcus with hoarseness, (2) sulcus with another laryngeal disease and (3) sulcus with no vocal disorder. The study found that 72% were male and that most of the patients were aged between 60 and 69 years old. The lesions were bilateral in 63% of the patients and unilateral in 37%, with no significant difference between the right and left sides. On the contrary, Sünter et al. [27] found that 56.4% of patients with sulci and benign vocal cord lesions were female, without reporting a significance difference in gender, with a mean age of 43.50 ± 12.7. In addition, in our study, most of the patients with sulci were females (67.53%) and were young, aged between 18 and 44 years old (58.44%), with a mean age of 42.09 ± 12.00. Varelas et al. suggest that the female prevalence may be due to anatomic and physiologic features. Indeed, women have smaller and thinner vocal cords and a higher F0 than men, meaning that they have increased fold vibration and, so, a higher risk to develop voice diseases due to phonotrauma [38]. Thus, the prevalence of women with SV in studies may be related to higher incidence of benign vocal fold lesions. Moreover, we found a statistically significant correlation between age and sulcus (*p* = 0.0005). Sünter et al. suggested that the young age of occurrence could be explained by a congenital sulcus [27].

However, regarding a possible correlation between the side and number of sulci and the side and number of vocal cord lesions, we did not find any statistical difference (*p* ≥ 0.05), as was reported by Itoh et al. [2]. Instead, in 2018, Carmel-Neiderman et al. [28] tried to detect a correlation between vocal fold polyps (VFP) and sulcus vocalis and found that patients with SV and VFP had a higher risk of developing contralateral vocal fold lesions due to phonotrauma (*p* = 0.04). A vocal polyp is a lesion of epithelium and a superficial layer of the lamina propria of the vocal fold; its onset is usually linked to vocal misuse and abuse due to possible pre-existing sulcus vocalis, resulting in phonotrauma. This assumption explains why the polyp usually develops on the same side as the SV.

In addition, in our study, the VFP was the most frequently found benign lesion in Group wSV (44.30%), although no statistically significant correlation was demonstrated.

Moreover, the study of Byeon et al. [31] supported our hypothesis that, in cases of the co-presence of SV and benign vocal fold lesions, the surgical treatment of the lesion without fixing the sulcus, may increase the risk of recurrence of the lesion itself. Indeed, in their study, the authors reported a significantly higher recurrence rate of VFP in the group with SV (16.7%) than in the group without SV (3.1%).

Moreover, in their casuistry, Sünter et al. [27] did not report the simultaneous presence of bilateral vocal nodules and SV. The authors explained that it is predictable since they have the same vocal fold localization, and SV usually causes phonotrauma around the sulcus itself. On the contrary, we found that 20.25% of Group wSV had vocal nodules without a statistical correlation with SV. Soares et al. stressed that most patients have bilateral sulcus vocalis (77%) [40]. The same results were observed by Yildiz et al., who found that 100% of included patients had bilateral SV [11]. In our study, we found a similar result: 40.26% of patients were affected by bilateral SV.

Nakayama et al. [33] studied sulcus vocalis in laryngeal cancer patients and demonstrated that sulci were more common in the cancer group, suggesting increased susceptibility; the chronic inflammation associated with tumors in adjacent or contralateral vocal folds might be a factor in the pathogenesis of sulcus vocalis. In our study, we excluded malignant glottic lesions because this kind of lesion could make it difficult to detect sulcus and, thus, impair statistical results as a bias.

As mentioned above, over the years, several therapeutic strategies have been proposed. In particular, there are surgical and non-surgical treatments. Voice therapy is indicated and effective in cases of type I sulci and consists of exercises to reduce vocal effort and to avoid compensatory mechanisms that may lead to vocal fold lesions. Pathological sulcus vocalis needs surgical strategies that include medialization laryngoplasty (also called type I thyroplasty) and vocal fold injections. In both cases, the main goal of the surgery is the medialization of the vocal cords to reduce the glottic incompetence. Up to now, vocal fold injections represent the best therapeutic approach with the filling of different materials: autologous fat, hyaluronic acid, steroids and/or platelet-rich plasma (PRP). Autograft fat is almost the best filler since it is inexpensive, biocompatible and easy-to-harvest. Moreover, a recent study demonstrated that vocal fold injections of PRP plus fat improve the advantages of fat, leading to faster post-operative vocal cord healing, longer lasting of the filler and softening of the cover layer. Indeed, the fat plus PRP mixture allows us to reach two goals: reduction in glottic insufficiency and reduction in vocal cord stiffness. Thus, the authors demonstrated that this type of vocal fold injections could be considered the best treatment for pathological sulcus since it allows us to achieve better vocal cord movement and vibration and a reduced glottic gap, with lasting results over time and a low risk of recurrence [41].

Tsunoda et al. [24] introduced a new technique consisting of the transplantation of autologous fascia into the vocal cords with SV. The authors formulated this technique based on the use of temporal fascia during tympanoplasty. Actually, this fascia improves vocal fold healing, and it is related to a low risk of infection and immunological reactivity. In this case, the temporal fascia is placed in a pocket, which is the Reinke’s space, between the cover and the body of the vocal cord. Thus, this surgery reduces the glottic gap and improves the mucosal wave during phonation.

Bouchayer et al. [1] suggested freeing the mucosa from the deeper layers of the vocal folds to restore the body-cover structure. Moreover, they suggested fixing the flap with fibrin glue. However, this so-called “epithelium freeing technique” does not allow us to overcome the glottic insufficiency, so this surgery should be associated with vocal fold injections.

However, this work has a few limits: first, it is a retrospective study, so it has less confidence and a lower level of evidence than prospective ones. In addition, we collected data from only one experienced surgeon; this could be a limit for data analysis, but this choice allowed us to avoid biases related to subjective operators’ skills. Indeed, as reported by Sünter et al. [26], the diagnosis of a sulcus is sometimes only made during a direct laryngoscopy with an experienced surgeon. In this regard, a recent study suggested the use of Narrow-Band Imaging (NBI) endoscopy to better detect SV in outpatients [42], as also shown in Figure 1 and Figure 2. Desuter et al. observed that, in cases of SV, the vessels that usually are parallel to vocal fold change their course and split, skirting the edges of the furrow. The authors called it “the lake road sign”, as well as the roads that surround lakes [43]. If this finding was the rule, NBI would be useful for SV diagnosis. Lim et al. reported capillary ectasis in 35% of patients with pathologic sulci, too, as a sign of severe inflammation [44]. In 1996, Ford et al. [6] found that mucosal vessels had a perpendicular course to the vocal folds in SV, suggesting that they may be considered “herald vessels” of pathologic sulci.

## 5. Conclusions

Sulcus vocalis is a longitudinal, full-thickness depression in the free edge of the true vocal fold that may impair phonation, causing phonotrauma. This question was the basis of the study: is there any cause–effect relationship between sulcus vocalis and benign vocal fold lesions? However, this study did not find any statistically significant correlation, except in the case of mild dysplasia, which may be due to chronic phonotrauma from sulcus itself. Moreover, we found that SV within a vocal fold lesion is more common in younger patients, suggesting a congenital nature of SV.

In conclusion, the take-home message is that in cases of benign vocal fold lesions, a possible SV should be considered and researched for two reasons: first, the surgeon should notify to patient that, in treating SV, the voice might change further, and, second, if the surgeon fails to heal the SV, the risk of recurrence of vocal fold lesion would be high.

However, further studies are needed to better know SV and its role in the onset of vocal fold lesions in order to provide the patient the best healthcare.

## Figures and Tables

**Table 1:** Characteristics of included patients and results of possible associations between variables.

Characteristics	Group wSV (%)	Group w/oSV (%)	Total (%)	Chi-Square (*p*-Value)
**Sex**				
*Female*	52 (67.53)	92 (60.53)	144 (62.88)	1.07 (0.299)
*Male*	25 (32.47)	60 (39.47)	85 (37.12)	
**Age**			46.61 ± 14.04 18–80	**14.92 (0.0005)**
*Mean ± SD*	42.09 ± 12.00	48.90 ± 14.44		
*Range*	18–74	18–80		
18–44 years old	45 (58.44)	55 (36.18)		
45–64 years old	30 (38.96)	72 (47.37)		
65–80 years old	2 (2.60)	25 (16.45)		
**Angioma**	1 (1.27)	0 (0)	1 (0.43)	-
Left	1	0	1	
Right	0	0	0	
Bilateral	0	0	0	
**Polyp**	35 (44.30)	53 (34.64)	88 (37.94)	2.42 (0.11)
Left	10	13	23	
Right	13	26	39	
Bilateral	12	14	26	
**Nodule**	16 (20.25)	27 (17.65)	43 (18.53)	0.304 (0.58)
Left	2	5	8	
Right	7	16	23	
Bilateral	6	6	32	
**Cyst**	7 (8.86)	14 (9.15)	21 (9.05)	0.0009 (0.97)
Left	3	7	10	
Right	4	6	10	
Bilateral	0	1	1	
**Reinke’s edema**	16 (20.25)	33 (21.57)	49 (21.12)	0.026 (0.87)
**Papillomatosis**	0 (0)	2 (1.31)	2 (0.86)	-
Left	0	0	0	
Right	0	1	1	
Bilateral	0	1	1	
**Keratosis**	3 (3.80)	4 (2.61)	7 (3.02)	0.275 (0.59)
Left	0	2	2	
Right	3	0	3	
Bilateral	0	2	2	
**Mild dysplasia**	**1 (1.27)**	**13 (8.50)**	**14 (6.03)**	**4.68 (0.03)**
Left	0	1	1	
Right	0	5	5	
Bilateral	1	7	8	
**Moderate dysplasia**	0 (0)	7 (4.57)	7 (3.02)	-
Left	0	1	1	
Right	0	3	3	
Bilateral	0	3	3	
**Total**	**79 (34.05)**	**153 (65.95)**	**232 (100)**	

There are 232 vocal fold lesions and 229 patients because we had 2 patients with a right nodule and a left cyst and 1 patient with a right polyp and a left nodule.

**Table 2:** Study of the correlation between the side of the lesion and the side of the sulcus.

Lesion Side				
Sulcus side	Right	Left	Bilateral	Total
Right	13	4	11	28
Left	5	4	9	18
Bilateral	4	7	20	31
**Total**	22	15	40	77
**Chi-square (*p*-value)**	8.2228 (0.08)			

## Data Availability

Not applicable.
